# Disease-Specific Survival of AJCC 8th Stage II Gastric Cancer Patients After D2 Gastrectomy

**DOI:** 10.3389/fonc.2021.671474

**Published:** 2021-07-26

**Authors:** Xiaohao Zheng, Yunzi Wu, Li Zheng, Liyan Xue, Zhichao Jiang, Chenfeng Wang, Yibin Xie

**Affiliations:** ^1^ Department of Pancreatic and Gastric Surgery, National Cancer Center/National Clinical Research Center for Cancer/Cancer Hospital, Chinese Academy of Medical Sciences and Peking Union Medical College, Beijing, China; ^2^ Department of General Surgery, The First People’s Hospital of Dongcheng District, Beijing, China; ^3^ Department of Pathology, National Cancer Center/National Clinical Research Center for Cancer/Cancer Hospital, Chinese Academy of Medical Sciences and Peking Union Medical College, Beijing, China; ^4^ Department of Medical Oncology, National Cancer Center/National Clinical Research Center for Cancer/Cancer Hospital, Chinese Academy of Medical Sciences and Peking Union Medical College, Beijing, China

**Keywords:** gastric cancer, disease-specific survival, prognosis, perineural invasion, distal gastrectomy, stage II

## Abstract

The association between the risk factors and long-term prognosis in patients with stage II gastric cancer after radical gastrectomy has been fully revealed. The purpose of this study was to investigate the independent risk factors for treatment failure in stage II gastric cancer. Demographic, clinical, and pathological information of 247 stage II gastric cancer patients who underwent radical D2 gastrectomy in our department between January 2011 and December 2014 were collected and retrospectively analyzed. The relationship between and long-term clinical outcomes of stage II gastric cancer was analyzed using t-tests, chi-square tests, receiver operating characteristic (ROC) analysis, time-dependent ROC analysis, K–M curves, and a Cox regression model. The median follow-up of 247 stage II gastric cancer patients was 5.49 years (range: 0.12–8.62 years). The Kaplan–Meier estimated 3-year and 5-year DSS rates of the study group were 92.7% (95% CI 89.4–95.9) and 88.7% (95% CI 84.7–92.7), respectively. Higher age (>70 *vs.* ≤70, log-rank p = 0.0406), nerve invasion (positive *vs.* negative, log-rank p = 0.0133), and non-distal gastrectomy (distal partial gastrectomy *vs.* other surgical methods, log-rank p = 0.00235) had worse prognoses compared to controls. Univariate and multivariate analyses of disease-specific survival showed that these three factors were independent prognostic factors for patients with stage II disease. The area under time-dependent ROC curve (AUC) is 0.748 of 5-year survival and c-index is 0.696 based on the three-marker model drawn for stage II patients. Subgroup analyses showed an interaction between tumor location and nerve invasion. The age, perineural invasion, and surgical approach are independent prognostic factors for disease-specific survival after radical gastrectomy. Tumor location may be an important confounding factor for outcomes by affecting surgical methods and the hazards of nerve invasion.

## Introduction

Gastric cancer is a common tumor, especially in East Asia, and remains the second leading cause of cancer-related deaths in China (1). As reported by the International Agency for Research on Cancer, nearly 1,000,000 people develop gastric cancer every year, and due to its poor prognosis, more than 760,000 people die of this disease each year (2). An advanced stage at the time of diagnosis is the main reason for high mortality, as more than 80% of gastric cancers could be cured if treated in a relatively early stage, including stages I and II. In recent years, stage I gastric cancer patients have received great attention because they can be treated by endoscopic resection with a 5-year disease-free survival rate of more than 90% (3). While other gastric cancer patients are observed to have a later stage of stage III or IV gastric cancer, approximately 70% of all gastric cancer patients in China also cause serious concern for their high mortality. Several clinical studies have been carried out in this population to compare the effects of different surgical techniques and chemotherapy regimens. Notably, stage II gastric cancer is rarely considered, and the treatment method is less studied. Although it is assumed that these patients have a relatively good prognosis, up to 30% of the patients with stage II gastric cancer relapse even after radical resection and adjuvant chemotherapy (4–6).

According to the 8th TNM staging manual of gastric cancer published by the Union for International Cancer Control/American Joint Committee on Cancer (UICC/AJCC), stage II gastric cancer consists of IIa (T1N2M0, T2N1M0, and T3N0M0) and IIb (T1N3aM0, T2N2M0, T3N1M0, and T4aN0M0) (7). As reported in large-scale studies, stage II accounts for approximately 20% of all gastric cancers, with a 5-year survival rate of 70% (4). The main reasons for treatment failure in relapsed patients who received D2 radical resection plus adjuvant chemotherapy remains unclear. Except for the TNM system, we usually consider tumor-related factors, such as worse differentiation, nerve invasion, blood vessel invasion, and treatment-related factors, such as laparoscopy or open surgery, excision extension, chemotherapy, or not as reasons for poor prognosis; however, studies only concentrating on stage II gastric cancer has rarely been carried out (8). To clarify the factors of poor prognosis in stage II gastric cancer and improve survival, we conducted this retrospective study in a single center.

## Materials and Methods

### Patients

In total, 1,514 gastric cancer patients were treated at the Cancer Hospital, Chinese Academy of Medical Sciences, from January 2011 to December 2014. We searched the clinicopathological database for primary gastric cancer patients with pathological TNM stage II, which were prospectively documented in the medical records. The inclusion criteria for this study were as follows: radical gastric gastrectomy with D2 dissection performed by senior surgeons according to the Japanese Gastric Cancer treatment guidelines; pathologically diagnosed as stage II gastric adenocarcinoma; no other malignant tumor history; postoperative survival time longer than 1 month; living patients with at least 3 years of follow-up time; pathological diagnosis report was confirmed by two or more senior pathological doctors; and sufficient oral intake and adequate organ function according to records at the first visit. Additionally, patients who received preoperative adjuvant chemotherapy were excluded because of their different pathological diagnosis criteria.

This retrospective study was approved by the Ethics Committee of Cancer Institute and Hospital, Chinese Academy of Medical Sciences, and the need for informed consent was waived.

### Treatment Methods

Treatment methods for all patients were decided by a multidisciplinary treatment (MDT) group including at least radiologists, pathologists, medical oncologists, and surgeons. According to contrast-enhanced chest–abdomen–pelvis computed tomography, upper gastrointestinal endoscopy, and judged by the MDT group, patients with clinical stage T1-3N0-2M0 were recommended to undergo surgical resection, and the need for postoperative adjuvant therapy was decided by medical oncologists according to the pathological reports and the results of immunohistochemical examination. Adjuvant chemotherapy with a regimen of S-1 plus oxaliplatin (SOX) was suggested for all pathological stage II patients. The resection extension of the gastric mucosa was decided by surgeons according to the treatment guidelines published by the Japanese Gastric Cancer Association (JGCA).

### Potential Risk Factors

Three aspects of parameters including the demographic information, such as age and sex, treatment-related factors, such as extension (distal gastrectomy, total gastrectomy, and proximal gastrectomy), minimally invasive or open approaches, adjuvant chemotherapy or not, tumor location (upper, middle, or lower third of the stomach), and pathological information, such as T stage, N stage, differentiation degree (well or poor), Borrmann type (0–4), Lauren type (mix, diffuse, or intestinal), vessel invasion (positive or negative), and nerve invasion (positive or negative) were included in this study as potential factors. Tumor-related factors were extracted from pathological reports that were made by two or more senior pathologists in the Department of Pathology, Cancer Hospital, Chinese Academy of Medical Sciences, according to the WHO guidelines. Two published nomograms for predicting disease-specific survival (DSS, Zhao et al.) (9) and OS (Zheng et al.) (10) were compared with the risk models in current dataset.

### Follow-Up

All patients were advised to undergo contrast-enhanced thoracic/abdominal/pelvic CT and blood testing every 3 months for the first 2 years and every 6 months thereafter. If the patients did not return to receive the follow-up examination at the scheduled time, the follow-up team of our hospital would contact them and record the reason.

### Statistical Analysis

The primary outcome was 5-year disease-specific survival (DSS, survival time from diagnosis to death from the specific disease). Categorical data were presented as absolute and relative frequencies, calculated using a chi-square test. The associations between these risk factors and DSS or relapse-free interval (RFI, interval from diagnosis to disease recurrence) were determined using the odds ratio (OR) and 95% confidence interval (CI) derived from logistic regression models. The Kaplan–Meier method was used to generate the survival curves. All statistical tests were two-sided, and a P-value less than 0.05, was considered as statistically significant. Analyses were performed using SAS software (version 9.4; SAS Institute, Cary, NC, United States).

## Results

Initially, 337 TNM stage II patients were extracted from the database, consisting of 22.3% (337/1,514) of all gastric cancer cases between January 2011 and December 2014. A total of 61 patients were lost to follow-up within 3 years. According to the inclusion/exclusion criteria, a total of 29 patients were excluded for tumor history (four cases), received preoperative chemotherapy (three cases), perioperative death (within 1 month in hospital, two cases), missing information in medical records (seven cases), and missing information in postoperative pathological records (13 cases). Finally, 247 TNM stage II gastric cancer patients (172 men, 75 women; mean age 57.5 years; range 25–81 years) were included in this study (**Table 1**). The median follow-up time of 247 patients was 5.49 years (range: 0.12–8.49 years). In total, 13.8% (34/247) of the patients were elderly (≥70 years), and the male to female ratio was 2.3. Notably, 64.0% (158/247) of the tumors were in the lower third part of the stomach, and 71.3% (176/247) of the operations were open surgery. For personal reasons, 19.4% (48/247) of the patients refused postoperative adjuvant chemotherapy. Pathological T2-3 (76.5%, 189/247) was most of the T stage, and N0-1 (80.2%, 198/247) was the predominant N stage. The TNM stages IIa and IIb were comparable (48.6% *vs.* 51.4%). Borrmann types 2–3 (81.7%, 201/246) were the most common macroscopic types. Positive vessel invasion and nerve invasion were observed in 35.4% (87/246) and 40.5% (100/247), respectively. Poorly differentiated tumors (81.0%, 200/247) were the most common.

**Table 1:** Demographic and clinical characteristics of the patients at baseline.

Variables	N (%)	Disease-specific survival rate (5-year, 95% CI)
**Age**		
≥70	34 (13.8)	75.8 (61.1–90.5)
<70	213 (86.2)	90.8 (86.9–94.8)
**Gender**		
Women	75 (30.4)	85.1 (76.9–93.2)
Men	172 (69.6)	90.4 (85.9–94.9)
**Tumor location**		
Upper	35 (14.2)	79.4 (65.7–93.1)
Middle	54 (21.9)	88.4 (79.6–97.1)
Lower	158 (64.0)	90.9 (86.4–95.5)
**Mini-invasive approach**		
Laparoscopy or thoracoscopy	71 (28.7)	89.5 (82.1–96.9)
Open approach	176 (71.3)	88.4 (83.6–93.2)
**Surgical ways**		
Transthoracic partial gastrectomy	6 (2.4)	66.7 (28.9–100)
Total gastrectomy	23 (9.3)	58.5 (37.4–79.5)
Distal gastrectomy	193 (78.1)	92.6 (88.9–96.3)
Proximal gastrectomy	25 (10.1)	91.4 (79.9–100)
**Adjuvant chemotherapy**		
With chemotherapy	199 (80.6)	89.3 (85–93.6)
Without chemotherapy	48 (19.4)	86.1 (75.7–96.5)
**Differentiation**		
Poorly differentiated	200 (81.0)	88.1 (83.6–92.7)
Well differentiated	47 (19.0)	91.5 (83.5–99.5)
**Pathological tumor stage**		
T4	37 (15.0)	83.4 (71.2–95.6)
T3	119 (48.2)	87.9 (81.9–93.9)
T2	70 (28.3)	91.2 (84.4–97.9)
T1	21 (8.5)	95.2 (86.1–100)
**Pathological nodal stage**		
N3a	6 (2.4)	83.3 (53.5–100)
N2	43 (17.4)	95.2 (88.8–100)
N1	91 (36.8)	87.2 (80.1–94.3)
N0	107 (43.3)	87.7 (81.4–94)
**TNM**		
IIb	120 (48.6)	86.1 (79.7–92.4)
IIa	127 (51.4)	91.2 (86.2–96.2)
**Macroscopic type**		
3-4	115 (46.7)	90.1 (84.5–95.7)
0-2	131 (53.3)	87.5 (81.8–93.2)
**Lauren type**		
Mix	64 (26.2)	91.8 (84.9–98.7)
Diffuse	102 (41.8)	90 (84.1–95.9)
intestinal	78 (32.0)	85.4 (77.4–93.4)
**Vessel invasion**		
Positive	87 (35.4)	89.5 (83.1–96)
Negative	159 (64.6)	88.3 (83.2–93.4)
**Perineural invasion**		
Positive	100 (40.5)	82.6 (75.1–90.2)
Negative	147 (59.5)	92.9 (88.7–97.2)

**Figure 1:**
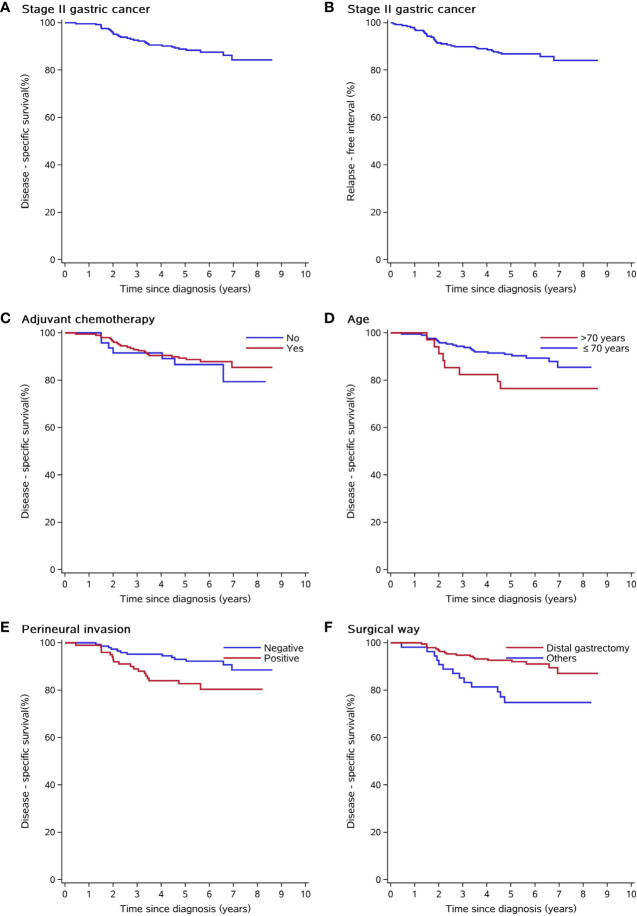
Kaplan–Meier curves of clinical and pathological risk factors. **(A)** Disease-free survival of the whole study group. **(B)** Relapse-free interval of the whole study group. **(C)** Disease-free survival was not different among patients who underwent adjuvant chemotherapy or not. **(D, E)** Elderly patients and patients with positive nerve invasion had shorter disease-specific survival. **(F)** Distal gastrectomy has better disease-specific survival than other surgical approaches (transthoracic partial gastrectomy, proximal gastrectomy, and total gastrectomy).

In total, 39 patients (15.8%, 39/247) died after surgery in the time range of 2 months to 7 years; additionally, eight patients died of cordis and cerebral accidents (n = 4), other tumors (n = 2), and intestinal obstruction (n = 2). At the time of the present follow-up, 31 patients (12.6%, 31/247) died of cancer relapse or distal metastasis. The Kaplan–Meier estimated 3-year and 5-year DSS rates of the study group were 92.7% (95% CI 89.4–95.9) and 88.7% (95% CI 84.7–92.7), respectively (**Figure 1A**). The Kaplan–Meier estimated 3-year and 5-year RFI rates of the study group were 89.8% (95% CI 86.1–93.6) and 86.7% (95% CI 82.4–91), respectively (**Figure 1B**). The DSS between the surgery alone group and D2 gastrectomy plus adjuvant chemotherapy (log-rank p = 0.526, **Figure 1C**). The patients of higher age (>70 years *vs.* ≤ 70 years, log-rank p = 0.0406, **Figure 1D**), nerve invasion (positive *vs.* negative, log-rank p = 0.0133, **Figure 1E**), and non-distal gastrectomy (distal partial gastrectomy *vs.* other surgical methods, log-rank p = 0.00235, **Figure 1F**) had worse prognoses compared to the controls.

Univariate and multivariate Cox analyses were performed to identify the clinical risk models related to DSS (**Table 2**). The results showed that age (>70 years *vs.* ≤70 years, HR 2.608, 95% CI 1.125–6.048), surgical method (distal gastrectomy *vs.* others, HR 0.458, 95% CI 0.218–0.962), and perineural invasion (positive *vs.* negative, HR 2.454, 95% CI 1.129–5.334) were independent risk factors for DSS of TNM stage II gastric cancer. The pathological tumor stage (pT) and pathological nodal stage (pN) did not significantly affect the prognosis (both p >0.05).

**Table 2:** Univariate and multivariable Cox analysis of disease-specific survival.

Parameter	Univariate				Multivariable			
	P-value	Hazard Ratio	95% Hazard Ratio Confidence		P-value	Hazard	95% Hazard Ratio Confidence	
Age in years (>70 *vs.*≤70)	0.0464	2.267	1.013	5.071	0.0255	2.608	1.125	6.048
sex (women *vs.* men)	0.5348	1.262	0.605	2.635				
Location (upper + middle *vs.* lower)	0.2345	1.536	0.757	3.116				
pT (T4a *vs.* T1–3)	0.4265	1.436	0.589	3.5				
pN (N2–3 *vs.* N0–1)	0.3089	0.58	0.203	1.657				
Vascular invasion (present *vs.* absent)	0.7855	0.901	0.424	1.914				
Stage (IIb *vs.* IIa)	0.3968	1.358	0.669	2.755				
Mini-invasive approach	0.8355	0.918	0.409	2.06				
Surgical way (distal *vs.* other)	0.0037	0.347	0.17	0.709	0.0393	0.458	0.218	0.962
Borrmann classification (3–4 *vs.* 0–2)	0.3171	0.691	0.335	1.425				
Differentiation (good *vs.* bad)	0.6718	1.23	0.472	3.206				
Lauren classification	0.8355	0.918	0.409	2.06				
Perineural invasion (present *vs.* absent)	0.0164	2.441	1.178	5.061	0.0234	2.454	1.129	5.334
Adjuvant chemotherapy (Yes *vs.* No)	0.5271	0.762	0.328	1.769				

**Figure 2:**
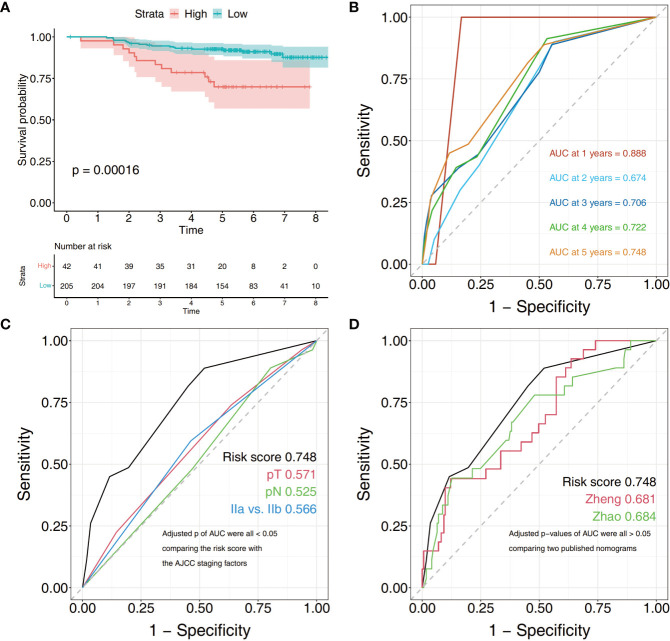
Kaplan–Meier survival analysis and ROC curves of the clinical factor signature in stage II gastric cancer cohort. **(A)** Kaplan–Meier curves for disease-specific survival of patients grouped by risk scores. **(B)** Time-dependent ROC analysis was performed to assess the prognostic accuracy, and P values were calculated using the log-rank test. **(C)** The risk score model has better prediction value than staging factors in stage II gastric cancer (pathological tumor stage, pathological nodal stage, and pathological AJCC stage). **(D)** Comparison of ROC curves of our risk model and others predicting survival. ROC, the receiver operating characteristic; AUC, area under curve.

A risk score formula was used to predict the DSS of stage II gastric patients as follows: risk score = −0.7808*(surgical method) + 0.8981*(perineural invasion) + 0.9590*(age). The use of 0.1782 as the cut-off value in patients in the high-risk group had a significantly worse prognosis compared with the low-risk group (**Figure 2A**). The area under the curve (AUC) values for time-dependent ROC analysis of the risk score model is plotted in **Figure 2B**. The time-dependent ROC analysis indicated that the AUC for the risk score signature was 0.888 (95% CI: 0.854–0.921) at 1 year, 0.674 (95% CI: 0.537–0.812) at 2 years, 0.706 (95% CI: 0.588–0.824) at 3 years, 0.722 (95% CI: 0.625–0.820) at 4 years, and 0.748 (95% CI: 0.632–0.844) at 5 years (**Figure 2D**). The c-index of the model is 0.696. The AUC of the risk score model is obviously of this signature was significantly larger than that of pathological tumor stage, pathological nodal stage, the AJCC stage (**Figure 2C**). The p values of the difference in the AUC between the risk score and staging factors are less than 0.05 (risk score *vs.*pathological tumor stage, p = 0.0381; risk score *vs.* pathological nodal stage = 0.005; risk score *vs.* AJCC staging subgroup, p = 0.021). We discovered that although the comprehensive model had the highest AUC value of 0.748 in the stage II cohort, there were no statistical differences between the risk score model and two well-accepted nomogram staging systems for DSS (Zhao et al., p = 0.657) and OS (Zheng et al., p = 0.558), respectively.

**Figure 3:**
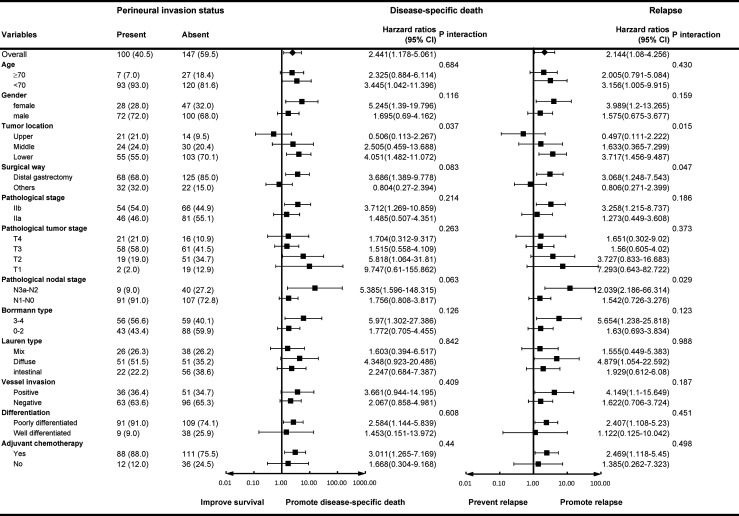
Subgroup forest analyses for nerve invasion. Hazard ratios for disease-specific death/relapse in the patients with nerve invasion are shown with 95% CIs.

In the subgroup forest plot (**Figure 3**), the results showed that nerve invasion in the lower third of the stomach tended to promote recurrence and disease-specific death than the upper and middle third parts (HR 3.717 for recurrence, 95% CI 1.456–9.487, p for interaction = 0.015; HR 4.051 for DSS, 95% CI 1.482–11.072, p for interaction = 0.037). In the surgical subgroup, perineural invasion in the distal gastrectomy group would promote recurrence compared to total gastrectomy (HR 3.068, 95% CI 1.248–7.543), whereas in the pathological N stage subgroup, the forest plot showed that perineural invasion in the N2–3a group would promote a relapse compared with the N0–1 group (HR 12.039, 95% CI 2.186–66.314).

## Discussion

There may arise a question regarding why we focused on stage II gastric cancer. Several studies have reported on the prognostic factors of gastric cancer, while in most of these studies, stages II and III were analyzed together. Additionally, most patients were stage III and the survival of this stage was poorer than stage II. In addition, stage II gastric cancer is in a relatively early stage and the disease is limited; hence, standard D2 radical gastrectomy plus adjuvant chemotherapy should cure these patients. Failure of treatment for this relatively early-stage disease should be considered.

The TNM staging system is the most important prognosis predicting method for gastric cancer, and currently, almost all therapeutic strategies are based on this system. While JGCA gastric cancer classification is also a widely accepted system representing the view of the east. Previously, there existed significant differences between the two systems, while now they have reached an agreement. Therefore, the importance of the TNM system is obvious. However, patients with the same TNM stage often have different clinical outcomes. In the same TNM stage II, about 70% of patients would survive without recurrence, while the other 30% patients would die of the disease; therefore, the TNM stage system is not enough by itself and needs assistant factors. This study aimed to identify the most effective factors for the additional judgment of prognosis in patients with stage II gastric cancer. The 3-factor risk panel has shown not only a significant advantage in stage II gastric cancer, and the non-inferior prediction efficacy compared with two published models with fewer variables.

Age was not an independent risk factor when the cut-off age was set at 60 years. However, if the cut-off was set at 70 years of age, elderly patients presented a significantly poor DSS. This may be because the basal metabolism of elderly people is lower and the oral intake tends to be inadequate, and constipation is common in the elderly population. Under the trauma of operation and insufficient intake caused by digestive tract reconstruction, the postoperative nutritional status and immunity function of elderly patients tend to be more dramatically impaired than in other populations. Current published nomograms or other type risk models often include the age, which may have a significant impact on patients’ long-term survival.

Like our results, several studies also showed that the tumor location of gastric cancer was an independent prognostic factor; additionally, the upper location was associated with worse prognoses. Recently, Ma et al. (2020) reported that the 5-year OS for patients with gastric cancer (stages I–III, 542 cases) located in the upper, middle, and lower third of the stomach were 35.0, 43.2, and 51.4%, respectively (11). With the tumor stage similar to Ma’s, some studies with similar results were published earlier. Yu et al. (964 cases) demonstrated that the 5-year OS rates were 28.0 and 51.0% for upper part gastric cancer and lower part gastric cancer patients, respectively (12). Liu et al. (439 cases) found that the 5-year OS rates were 27.4 and 49.5% for upper and lower gastric cancer patients, respectively (13). Kim et al. (2,696 cases) reported that the 5-year OS for gastric cancer patients located in the upper third and the middle third/lower third was 49.3 and 57.3%, respectively (14). Even in stage IA, a recent retrospective study of 1,707 cases of clinical T1N0 gastric cancer patients also showed that the prognosis of the patients with cancer in the upper third of the stomach was significantly worse than that of the patients with cancer in the middle or lower third groups (15). The exact reason for the survival difference among upper, middle, and lower locations remains unclear; however, a hypothesis exists. This may be due to the lack of visceral peritoneum for the intra-abdominal part of the cardia and fundus, which would make the tumor infiltrate the serosa more easily (16) or the plenty of large autonomic nerves in the upper third of the stomach, which provides a path for the spread of the tumor (17).

Several studies have been performed on the perineural invasion (PNI) of gastric cancer. Hwang et al. (18) reported that PNI accounted for 42.7% of all patients, which is similar to the results of the present study (40.5%). However, the incidence of PNI in the tumors of gastric cancer patients varied dramatically according to the reports of different institutions, from the possibly highest 75.6% (19) to the lowest (less than 10%) (20). The detection method and the experience of pathologists may have contributed to this difference. Previous studies have shown that PNI is strongly associated with a number of unfavorable prognostic factors, such as larger tumor size, vessel invasion, worse differentiation, advanced T and N stage, and so on. Hence, PNI could be designated as a predictive prognostic factor for gastric cancer patients and researchers have proved that if PNI is incorporated into the TNM staging system, the prognosis of stage III gastric cancer patients would be more accurately predicted. The results of the present study showed that although the survival of stage II lower part gastric carcinoma was relatively better, PNI tended to promote recurrence and decrease DSS (HR3.717 for recurrence, 95% CI 1.456–9.487, P interaction = 0.015; HR4.051 for DSS, 95% CI 1.482–11.072, P interaction = 0.037). Especially in stage II gastric cancer patients with advanced N stage (N2–3a), PNI was a stronger predictor of disease recurrence (HR 12.039, 95% CI 2.186–66.314, P interaction = 0.029).

Concerning postoperative chemotherapy, the inconsistency of our results with those of two previous large-scale phase III clinical studies showed some limitations of retrospective studies (5, 21). In the present study, chemotherapy was routinely recommended to all patients with stage II gastric cancer, while 19.4% (48/247) of the patients refused postoperative chemotherapy for personal reasons, with a 5-year DSS 86.1% slightly lower than those who received chemotherapy (5-year DSS 89.3%). However, regarding the PNI and tumor location, our results accorded with most of the previous studies, and at the same time, we found that elderly patients with stage II gastric cancer might show worse survival.

This study has limitations as it is a retrospective study with small sample size and different treatment protocols may have been applied. A sample size that is too small increases the likelihood of a Type II error, which decreases the power of the study. Some minor but valuable risk factors may be overlooked. Moreover, the cohort were presented in a high-level center with relatively better treatment results, and is not the representative of the general population. Our results need to be further confirmed by large-scale prospective randomized studies.

## Data Availability Statement

The raw data supporting the conclusions of this article will be made available by the authors.

## Ethics Statement

This retrospective study was approved by the Ethics Committee of Cancer Institute and Hospital, Chinese Academy of Medical Sciences, and the need for informed consent was waived.

## Author Contributions

XZ and YX contributed to study conception and manuscript writing. XZ, LZ, LX, and YW contributed to data collection and analysis. YX, CW, and ZJ contributed to clinical treatment. All authors contributed to the article and approved the submitted version.

## Funding

The study was supported by the CAMS Initiative for Innovative Medicine (2016-I2M-1-007) and China International Medical Foundation (CIMF-F-H001-314).

## Conflict of Interest

The authors declare that the research was conducted in the absence of any commercial or financial relationships that could be construed as a potential conflict of interest.

## Publisher’s Note

All claims expressed in this article are solely those of the authors and do not necessarily represent those of their affiliated organizations, or those of the publisher, the editors and the reviewers. Any product that may be evaluated in this article, or claim that may be made by its manufacturer, is not guaranteed or endorsed by the publisher.
